# Supplementary material for: Investigating microbial population structure and function in the chicken caeca and large intestine over time using metagenomics
Source: BMC Res Notes. 2025 Aug 15;18:355. doi: 10.1186/s13104-025-07441-7 (PMC12357378; doi:10.1186/s13104-025-07441-7)
Supplement: Supplementary file 2 — Recovered nutrient cycles including Carbon, Nitrogen, and Sulphur cycles for all the metagenomic assembled genomes given as PDF images, and identifiable through bin numbers. [file 13104_2025_7441_MOESM2_ESM.zip › Supplementary Data2/bin.139.draw_sulfur_cycle_single.pdf]

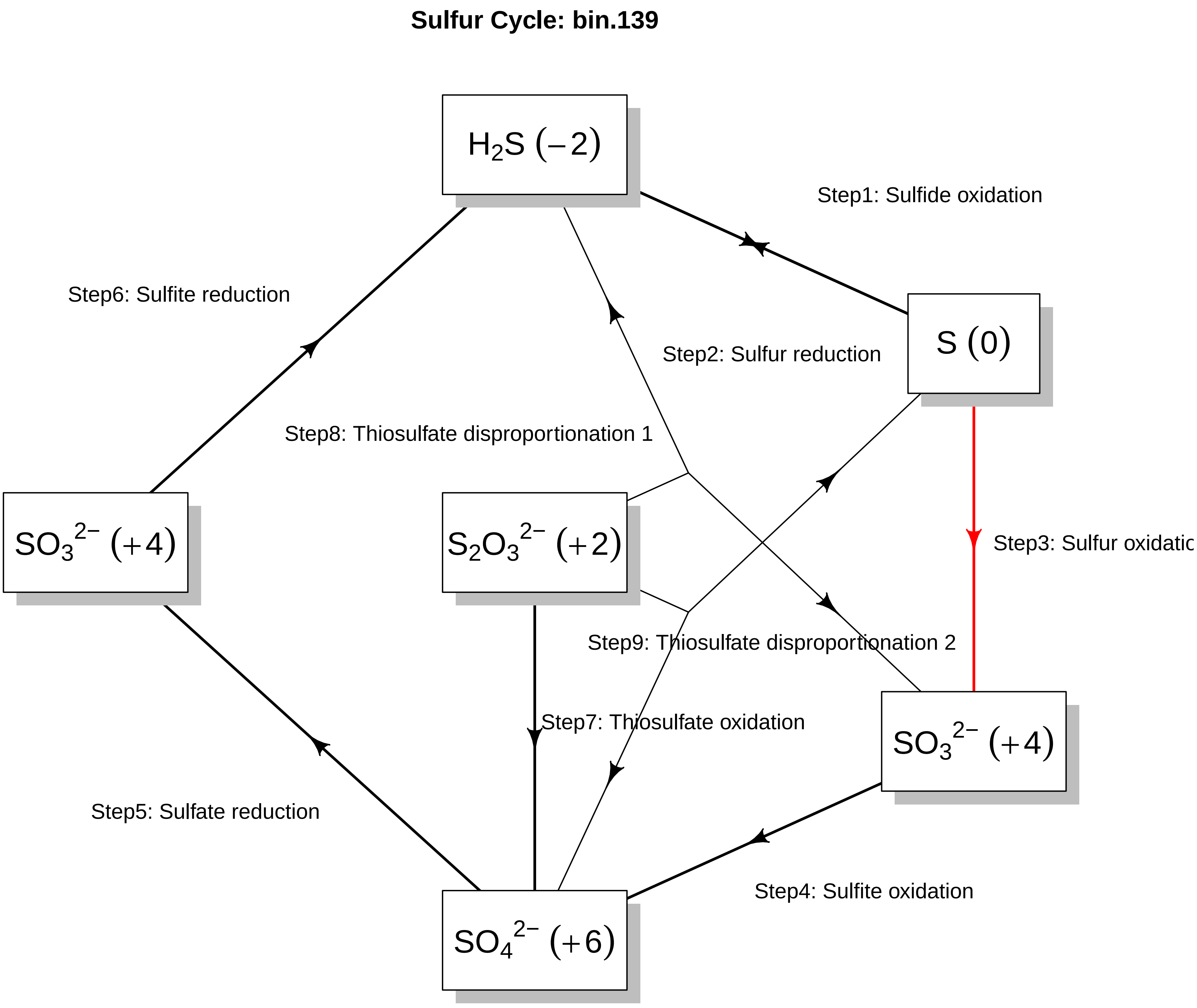
Sulfur Cycle: bin.139
H2S (−2)
S (0)
SO3 2− (+4)
S2O3 2− (+2)
SO3 2− (+4)
SO4 2− (+6)
Step1: Sulfide oxidation
Step2: Sulfur reduction
Step3: Sulfur oxidatic
Step4: Sulfite oxidation
Step5: Sulfate reduction
Step6: Sulfite reduction
Step7: Thiosulfate oxidation
Step8: Thiosulfate disproportionation 1
Step9: Thiosulfate disproportionation 2
